# Disulfide Cross-Linked Polymeric Redox-Responsive Nanocarrier Based on Heparin, Chitosan and Lipoic Acid Improved Drug Accumulation, Increased Cytotoxicity and Selectivity to Leukemia Cells by Tumor Targeting via “Aikido” Principle

**DOI:** 10.3390/gels10030157

**Published:** 2024-02-20

**Authors:** Igor D. Zlotnikov, Alexander A. Ezhov, Natalia V. Dobryakova, Elena V. Kudryashova

**Affiliations:** 1 Faculty of Chemistry, Lomonosov Moscow State University, Leninskie Gory, 1/3, 119991 Moscow, Russia; zlotnikovid@my.msu.ru (I.D.Z.);; 2 Faculty of Physics, Lomonosov Moscow State University, Leninskie Gory, 1/2, 119991 Moscow, Russia

**Keywords:** glutathione sensitivity, tumor targeting, polymeric micelles, doxorubicin, non-target toxicity

## Abstract

We have developed a micellar formulation of anticancer drugs based on chitosan and heparin grafted with lipoic and oleic acids that can release the cytotoxic cargo (doxorubicin) in response to external stimuli, such as increased glutathione concentration—a hallmark of cancer. Natural polysaccharides (heparin and chitosan) provide the pH sensitivity of the nanocarrier: the release of doxorubicin (Dox) is enhanced in a slightly acidic environment (tumor microenvironment). Fatty acid residues are necessary for the formation of nanoparticles (micelles) and solubilization of cytostatics in a hydrophobic core. Lipoic acid residues provide the formation of a labile S-S cross-linking between polymer chains (the first variant) or covalently attached doxorubicin molecules through glutathione-sensitive S-S bridges (the second variant)—both determine Redox sensitivity of the anticancer drugs carriers stable in blood circulation and disintegrate after intracellular uptake in the tumor cells. The release of doxorubicin from micelles occurs slowly (20%/6 h) in an environment with a pH of 7.4 and the absence of glutathione, while in a slightly acidic environment and in the presence of 10 mM glutathione, the rate increases up to 6 times, with an increase in the effective concentration up to 5 times after 7 h. The permeability of doxorubicin in micellar formulations (covalent S-S cross-linked and not) into Raji, K562, and A875 cancer cells was studied using FTIR, fluorescence spectroscopy and confocal laser scanning microscopy (CLSM). We have shown dramatically improved accumulation, decreased efflux, and increased cytotoxicity compared to doxorubicin control with three tumor cell lines: Raji, K562, and A875. At the same time, cytotoxicity and permeability for non-tumor cells (HEK293T) are significantly lower, increasing the selectivity index against tumor cells by several times.

## 1. Introduction

In modern biomedicine, one of the urgent tasks is to create targeted drug delivery systems [1,2,3,4,5,6,7]; this is especially true for oncological diseases, which require long-term treatment with high dosages. Therefore, in order to improve the quality of life, it is important to find optimal and effective methods of chemotherapy [8]. Existing medical strategies mostly have one common drawback—high non-target toxicity (especially heart, liver, and kidneys) [9,10]. To increase the selectivity of cytostatics (on the doxorubicin model) against tumors, we developed drug delivery systems based on polymeric nanoparticles (micelles). A key aspect of these delivery systems is the presence of a trigger on target cells, in this work—tumor cells [11,12,13,14,15,16,17,18]. Besides the approaches based on actively targeting tumor cells via specific receptors (folic and sialic acid residues, biotin, antibodies, peptides, glucose transporters [19]), in the case of tumors, the following differences from normal tissues can be taken into attention [6,19,20,21,22,23,24,25,26,27,28,29,30,31,32]: (1) a slightly acidic environment (pH 5.5–6.5), (2) a local increase in temperature, (3) an increase in blood viscosity (including local thrombosis), (4) altered morphology of cancer cells and increased permeability (leaky membrane), and (5) increased concentrations of reduced glutathione (GSH).

“Smart” polymeric micelles can specifically respond to certain triggers, for example, pH, temperature, radiation, Redox potential, ionic strength, and biological stimuli [33]. The creation of “smart” polymeric nanogel particles makes it possible to increase the effectiveness and reduce the toxicity of antitumor drugs [22,26,31,32,34,35]. In this paper, we developed stimuli-sensitive «smart» polymeric micelles based on chitosan or heparin grafted with fatty acid with the function of tumor targeting and delivery of the model cytostatic doxorubicin (Dox).

The pH sensitivity in polymeric micelles is provided by chitosan [26], a biocompatible, biodegradable polymer with a p*K*_a_ of the amino group of the order of 6.2–6.4 units. In a weakly acidic medium corresponding to the tumor microenvironment, chitosan amino groups are protonated, and the micelle structure is loosened with the drug release. This effect is especially evident when the drug molecule itself is similarly charged. For example, Dox is positively charged at pH < 8, which causes repulsion from polymer chains in the tumor medium.

Thermal sensitivity in smart particles is provided by polymer chains (thermally dependent gels or thermogels, hydrogels [36,37,38,39]) of chitosan or heparin, which undergo changes in the microstructure with an increase in temperature from 37 to 40–42 °C.

Also, Redox sensitivity, namely glutathione (GSH) sensitivity of polymeric nanoparticles, ensures the presence of labile disulfide bonds between polymer chains or between polymer and drug. GSH is the most important antioxidant in cells [40,41]. GSH is found in all cell compartments in millimolar concentrations (1–10 mM). In the case of cancer, GSH plays both a protective and pathogenic role. It is involved in the detoxification of carcinogens, and changes in this pathway can have a profound effect on cell viability. An increased concentration of GSH accumulates in cancer cells, which may cause resistance to antitumor drugs (cytostatics). «Smart» micelles use this feature of cancer cells: GSH as a trigger causes accelerated release of cytostatic.

An additional effect of polymeric nanoparticles on the chemotherapy effectiveness can be expected due to heparin. Patients with oncological diseases have a significantly increased risk of micro-thrombosis [42,43,44], which secondarily provokes problems with the cardiovascular system, brain, and thromboembolism. Indeed, cancer determines the activation of chronic coagulation due to the production of procoagulant substances by tumor cells (tissue coagulation factor, cysteine transpeptidase, etc.), which increases thrombotic activity; moreover, idiopathic thromboembolism (Trousseau’s syndrome) is often associated with cancer. It has also been shown that in the presence of a number of tumor cell lines, the antithrombotic activity of heparin (antithrombin activator) is neutralized [45]. Therefore, we suggest using the antithrombotic agent heparin as the main component of polymeric micelles to reduce blood viscosity and prevent the risk of thrombosis [45,46,47,48].

The other important differences between cancer cells and normal ones are the following morphological features [49]: an enlarged nucleus, an increased ratio of nucleus and cytoplasm, altered membrane, hyperchromasia, and abnormal chromatin distribution—in other words, cancer cells are “defective” and this can be used to deliver targeted drugs.

Currently, the use of “smart” delivery systems for the treatment of oncological diseases will increase the effectiveness of therapy for various types of cancer, including leukemia. Complex therapy is used in the treatment of acute lymphoblastic leukemia (ALL) [50,51,52,53,54,55]: doxorubicin, Vincristine, Methotrexate, Glucocorticoid (prednisone or dexamethasone), in combination with L-asparaginase enzyme-therapy. Treatment protocols include combinations of different drugs at each stage to minimize the risk of drug resistance and increase the likelihood of cure. Therefore, micellar formulations of Dox in combination with L-asparaginase represent promising ways to treat leukemia.

For a comprehensive study of “smart” drug delivery systems, we studied two fundamentally different types of cells: (i) leukemia cells K562 and Raji lymphoma cells (i.e., blood cells with phagocytotic activity, presumably they will absorb micellar particles with Dox) in comparison with (ii) skin cancer cells A875 (as a control type, non-phagocytic epithelial cells). In this way, we could compare the permeability of different types of cells for Dox, non-covalent, and covalent micellar formulations based on chitosan or heparin and correlate with antitumor activity. In this paper, the key idea is to increase the effectiveness and selectivity of the cytostatic drug by taking advantage of the differences in morphology and metabolism of tumor cells against themselves, i.e., implementing the so-called “Aikido principle” using the “smart” polymer nanoparticles. Thus, the creation of the drug carrier is stable in blood circulation and disintegrates after intracellular uptake by tumor cells. Therefore, here we aimed at the development of stimulus-sensitivity “smart” delivery systems where the characteristics of cancer cells are used to target them. In such a way, the “Aikido” principle is realized: the use of the strength of the enemy against itself. This will potentially increase the effectiveness of chemotherapy and reduce the systemic burden on the organism.

## 2. Results and Discussion

### 2.1. The Synthesis and Characterization of Amphiphilic Polymers and Dox-Containing Micelles

#### 2.1.1. Heparin and Chitosan Micelles

The concept is based on targeted delivery to tumors of the drug loaded in polymeric micelles. Tumor targeting is realized due to the pH-, thermo- (we shown earlier [26]), and glutathione-sensitivity of the micelles based on heparin and chitosan grafted with fatty acids (oleic and lipoic). We suggested polymeric micelles as drug delivery systems due to their dual nature: (1) the ability to incorporate drug molecules into the hydrophobic core, thereby solubilizing drug and protecting it from destruction, as well as drug ingestion into non-target cells and tissues; (2) biocompatible polymers are harmless to the body, and at the same time heparin has antithrombotic properties (thrombosis is increased in tumors) [56], chitosan has pH-sensitivity due to protonation of amino groups in a weakly acidic medium corresponding to the microenvironment of tumors.

To create an optimal delivery system, we studied both cationic and anionic polymers, as well as covalent and non-covalent micellar formulations with Dox. Table 1 shows the designations and characteristics of the developed polymers and micelles.

The critical micelle concentration (CMC) values for conjugates and polymeric micelles in the presence of Dox in comparison with Dox-free systems using a pyrene-based probe show that the drug loading with Dox had a negligible effect on the micelles formation.

#### 2.1.2. The Synthesis of Amphiphilic Polymers and Dox Prodrugs

**Figure 1:**
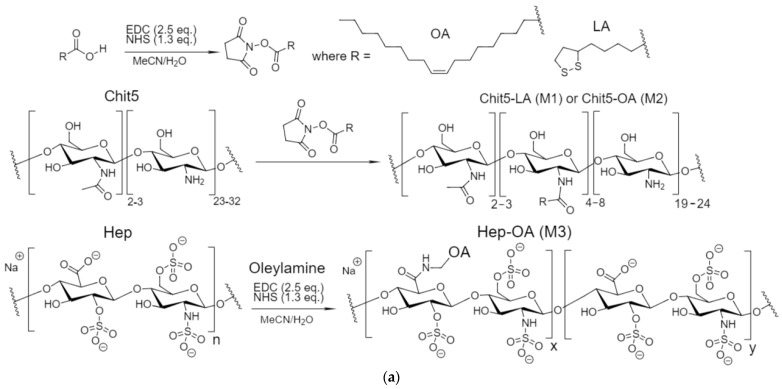

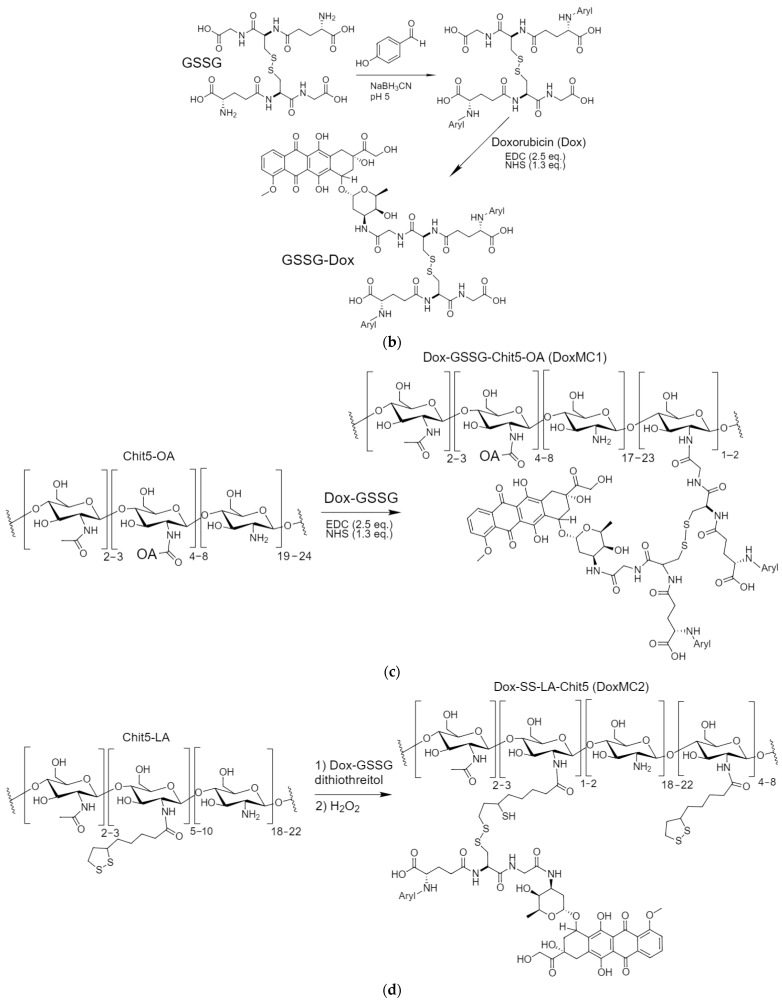
The schemes of synthesis of (**a**) amphiphilic conjugates Chit5-LA, Chit5-OA, and Hep-OA; (**b**) glutathione-sensitive doxorubicin *Dox-GSSG*; (**c**) covalent Dox conjugate Dox-GSSG-Chit5-OA (*Dox MC1*); (**d**) covalent Dox conjugate Dox-SS-LA-Chit5 (*Dox MC2*).

To obtain micelles, firstly, amphiphilic polymers based on polycations or polyanions and hydrophobic substituents were synthesized. The schemes for chitosan and heparin grafted with fatty acids are shown in Figure 1a. The idea of synthesis is the activation of the carboxyl group of lipoic or oleic acids with subsequent cross-linking with the chitosan amino group using a carbodiimide approach. In the case of heparin, the situation is the opposite: the carboxyl group of heparin and the amino group of oleylamine are cross-linked using the same approach.

Obtaining covalent prodrugs based on Dox is a more cunning way. It is worth considering here that it is necessary to obtain not just a covalent cross-linking of the Dox-polymer but a labile bond with the possibility of destruction only in the tumor microenvironment—this is a disulfide bond. Here, we obtained the Dox–glutathione conjugate (Dox-GSSG, Figure 1b), which was used for the subsequent obtaining of prodrugs (Figure 1c,d). DoxMC1: doxorubicin is attached to the polymer through cross-linked glutathione residues with an S-S bond. DoxMC2: doxorubicin is attached to the polymer through glutathione residue and lipoic acid stitched with an S-S bond.

#### 2.1.3. FTIR Spectroscopy for Characterization of Self-Assembled Chitosan and Heparin Conjugates

**Figure 2:**
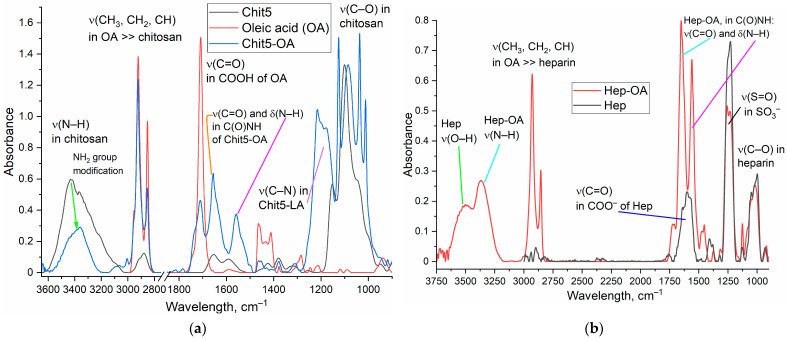
FTIR spectra of (**a**) Chit5, OA, its conjugate Chit5-OA; (**b**) Hep, Hep-OA. PBS (0.01 M, pH 7.4). T = 22 °C.

FTIR spectroscopy is one of the key methods for analyzing the molecular architecture and chemical structure of biological polymers since such systems are complex and heterogeneous, which complicates the analysis by other classical methods such as mass spectrometry and NMR (are given in Supplement) spectroscopy. On the contrary, the IR spectra of biopolymers and conjugates are quite compliant to analyze and provide valuable information. Figure 2 shows FTIR spectra of chitosan (Chit5), oleic acid (OA), and Chit5-OA and Hep-OA conjugates. The spectra of OA and conjugates contain characteristic bands of valence oscillations of CH_2_ groups in oleic residues (2980–2850 cm^−1^). When chitosan is modified by oleic acid residues, the formation of an amide group C(=O)NH occurs from carboxylic group COOH, respectively, the intensity of the peak at 1710 cm^−1^ decreased, and two peaks appear at 1660 and 1560 cm^−1^ (Figure 2a). A part of chitosan amino groups modified into an amide cross-linking; therefore, the intensity of the NH oscillation band 3600–3200 cm^−1^ decreased. The grafting of chitosan with fatty acids leads to a change in the shape and structure of the peak of C–O–C bond oscillations (1200–1000 cm^−1^) in the chitosan polymer chain: the two-component peak became multicomponent due to the different hydrophobicity of the microenvironment of glucosamine residues.

In the FTIR spectra (Figure 2b) of heparin (Hep) and its conjugate with oleylamine, high-intensity bands corresponding to sulfogroups (1250 cm^−1^), as well as C–O–C oscillation band (1100–1000 cm^−1^) are observed. Modification of heparin occurs due to the formation of an amide bond C(=O)NH, which is reflected in the IR spectra: two peaks appear at 1660 cm^−1^ (valence C=O) and 1560 cm^−1^ (deformational N–H) (Figure 2b). In addition, a peak corresponding to the valence oscillationы of N–H in the amide bond appears in the spectrum of the He_3_-OA conjugate compared to the Hep spectrum. Thus, FTIR spectroscopy confirms the chemical composition of amphiphilic polymers based on chitosan and heparin.

#### 2.1.4. FTIR Spectroscopy for Characterization of Dox-Containing Micellar Formulations

The main objective of the work is to obtain cytostatic micellar formulations selective for tumors. An important parameter for the realization of selectivity is pH and glutathione sensitivity. The first is realized due to chitosan; the second is due to disulfide bonds between polymer chains of amphiphilic conjugates in the micelle (polymer-polymer) or disulfide bonds Dox-polymer. These are two fundamentally different strategies for glutathione sensitivity since covalent modification of the drug, on the one hand, will increase the target bioavailability but, at the same time, may reduce the activity of the drug. Therefore, it is advisable to study both non-covalent micellar formulations based on Dox (*DoxM* series) and covalent Dox-polymer formulations (*DoxMC* series).

**Figure 3:**
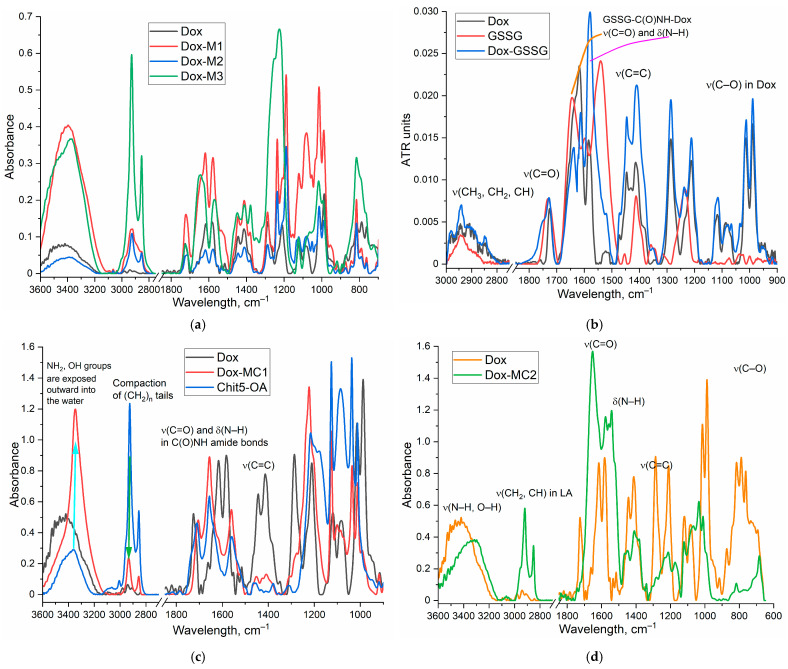
FTIR spectra of (**a**) Dox and its non-covalent micellar formulations Dox M1, M2, M3; (**b**) Dox, oxidized glutathione GSSG and its conjugate Dox-GSSG; (**c**) Dox and its covalent conjugate with Chit5-OA (Dox MC1); (**d**) Dox and its covalent conjugate with Chit5-LA (Dox MC2). PBS (0.01 M, pH 7.4). T = 22 °C.

*DoxM* series. The FTIR spectra of Dox and non-covalent DoxM1-M3 formulations are shown in Figure 3a. All characteristic peaks of Dox are present in the spectra of micellar formulations: 1725 cm^−1^ (ν(C=O)), 1611 and 1582 cm^−1^ (δ(N–H)), 1445 and 1414 cm^−1^ (ν(C=C)), and 1015 and 988 cm^−1^ (ν(C–O)). The inclusion degree of Dox in chitosan micelles (M1-M2) is about 10–13%, and in heparin micelles is about 6% (Table 1). The inclusion of Dox in micelles is additionally confirmed by the shifts of the peak maximum in the IR spectra: (1) the peak at 1414 cm^−1^ shifts to 1411–1412 cm^−1^, which indicates an increase in the hydrophobicity of the microenvironment of the aromatic Dox system in the core of micelles; (2) the peak of the carbonyl group oscillations shifts from 1724 to 1718–1721 cm^−1^, which indicates a decrease in the hydration degree of the C=O group, that is an increase in the hydrophobicity of the microenvironment of the cytostatic molecule.

*DoxMC* series. The FTIR spectra of Dox and covalent polymeric formulations are shown in Figure 3b–d. In the FTIR spectrum of the conjugate of Dox and glutathione (GSSG) (Figure 3b), bands at 1640 and 1580 cm^−1^ appear, corresponding to oscillations in the formed amide bonds of Dox-C(=O)NH-GSSG, as well as characteristic bands of both components. The synthesized Dox-GSSG conjugate was covalently attached via an amide bond to the Chit5-OA polymer (Figure 3c) and, using thiol-disulfide exchange agents, was attached via a labile S-S bond to lipoic acid residue in Chit5-LA polymer (Figure 3d and Figure S2). The success of the synthesis is confirmed by the FTIR spectra in a similar way to the above reasoning: by the presence of peaks of all components and the appearance of peaks amide 1 and amide 2 after cross-linking. However, there are individual features for DoxMC1 and DoxMC2 conjugates. The formation of micelles from DoxMC1 polymers was accompanied by the formation of a hydrophobic core and the compaction of (CH_2_)_n_ tails (Figure 3c), while hydrophilic NH_2_, OH groups are exposed outward into the water. For DoxMC2-based Dox-containing micelles, the key parameter is the presence of labile S-S bonds, which is confirmed by a decrease in the peak intensity of the corresponding oscillations of the S-H groups (Figure S2—2580, 2495 cm^−1^) due to cross-linking. Thus, we have successfully synthesized non-covalent micellar Dox-containing formulations and covalent Dox-polymer conjugates.

#### 2.1.5. Atomic Force Microscopy for Micelles Visualization and Morphology Characterization

**Figure 4:**
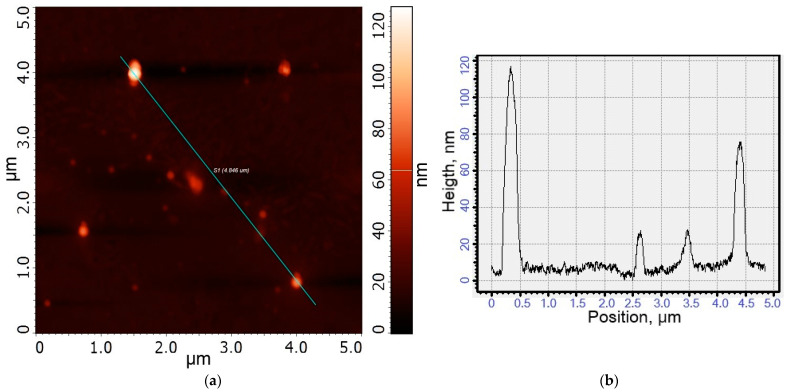

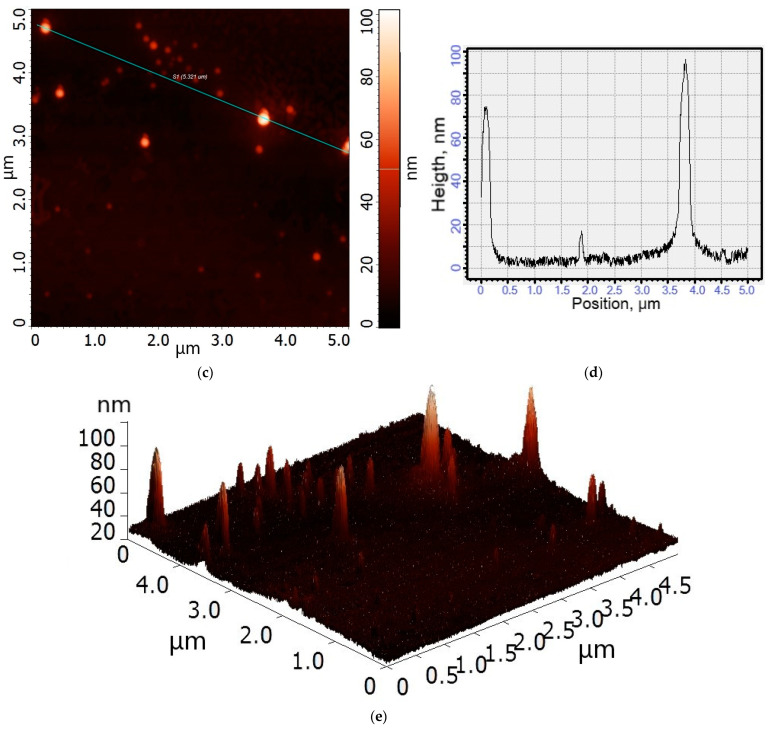
Atomic force images of micelles formed from (**a**) Dox-MC1 and (**c**) Dox-MC2 conjugates. (**b**,**d**) The corresponding height profiles of (**a**,**c**). (**e**) 3D image of Dox-MC2 micelles. The surface is freshly ground mica.

Using FTIR spectroscopy, we confirmed the successful synthesis of polymers and conjugates with Dox. Atomic force microscopy (AFM) was used to demonstrate the micelle-forming properties of these polymer conjugates. Figure 4 shows images of the micelles formed by amphiphilic covalent conjugates of Dox. The average size of the Dox-MC1 micelles is approximately 120–200 nm, while the Dox-MC2 micelles are about 130–180 nm. This difference is due to the fact that oleic acid residues are larger than that of lipoic acid, and, in addition, the latter can form disulfide bonds that seal the structure of the micelle. Unlike non-covalent micellar formulations previously described in work [26,57,58], covalent conjugates retain their spherical shape well when applied to a substrate (Figure 4e), providing polymer micelles loaded with Dox.

#### 2.1.6. Hydrophobic–Hydrophilic Balance in Polymeric Micelles

Fluorimetry using a pyrene probe was employed to investigate the hydrophobic–hydrophilic balance within polymer micelles (Figure S4). The pyrene molecule can exist in two forms: a monomer (emission at 380–400 nm) in a hydrophilic environment and an excimer (emission at 460 nm) in a hydrophobic environment. The hydrophobic microenvironment of pyrene is manifested in micelles formation, indicating the aggregation and compactization of the polymers with the formation of the micellar core.

### 2.2. Glutathione-Sensitivity of Conjugates

A key parameter for selective drug delivery to tumors is stimulus-sensitivity to the tumor cells microenvironment. Stimuli can be understood as (1) a slightly acidic environment (pH 5.5–6.5), (2) a local increase in temperature, (3) an increase in blood viscosity (thrombosis), (4) increased concentrations of reduced glutathione GSH. We examined the sensitivity of chitosan micelles to pH and temperature in a separate paper [26] and showed that (1) at pH 5.5–6 (tumor microenvironment model), the rate of Dox release is 2–3 times higher than at pH 7–7.4; (2) Dox release rate was increased up to 2 times with a temperature increase from the physiologically relevant 37 °C to local inflammatory zone with 42 °C. We investigated the third aspect regarding antithrombotic drug delivery systems to tumors, since we developed the heparin-based polymeric micelles, since heparin is an antithrombin activator [45,46,47,48].

**Figure 5:**
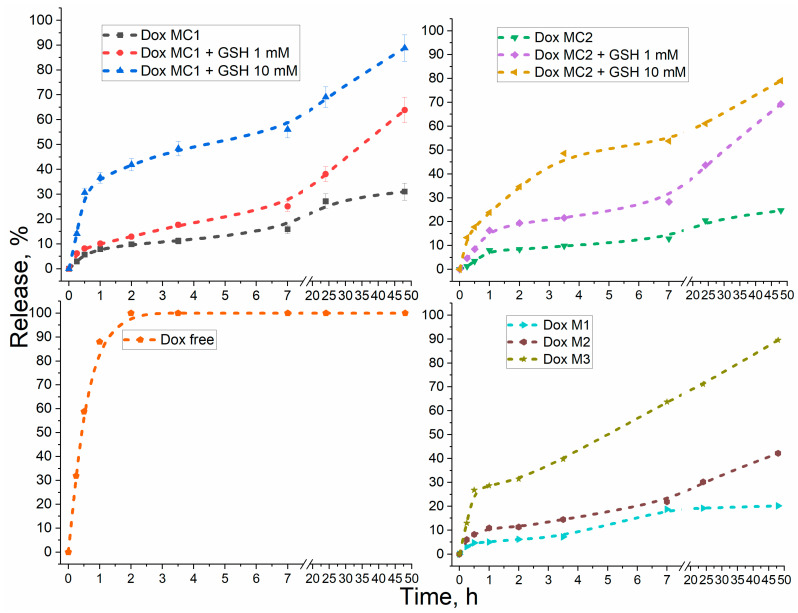
Dox release kinetic curves for different formulations: Dox-free, Dox non-covalent micellar formulations, and Dox covalent conjugates in the presence of 0/1/10 mM reduced glutathione (GSH). PBS (0.01 M, pH 7.4). T = 37 °C. DoxM1—Dox in Chit5-LA, DoxM2—Dox in Chit5-OA, DoxM3—Dox in Hep-OA, DoxMC1—Dox-GSSG-Chit5-OA, DoxMC2—Dox-SS-LA-Chit5.

Finally, the most important aspect is glutathione sensitivity, that is, the ability of micelles to release the drug at elevated GSH concentrations (up to 20 mM). At the same time, the difference in the concentration of glutathione (GSH) inside cells (2–4 mM, in tumor cells 4–5 times higher to 20–30 mM) and in extracellular fluid (2–20 μM). We have developed non-covalent micellar formulations with Dox (DoxM1-M3), which significantly slow down the rate of Dox release (Figure 5, Table 2) compared with free drug; however, the nature of this release is not glutathione-dependent. At the same time, covalent conjugates DoxMC1 and DoxMC2 demonstrate an increase in the initial rate of Dox release up to 6 times in the presence of 10 mM GSH, while in 7 h, the accumulated concentration of Dox is 5–6 times higher in the model tumor microenvironment. Thus, the covalent conjugates DoxMC1-MC2 are glutathione-sensitive and able to selectively release the drug in the tumor microenvironment.

### 2.3. Cytotoxicity Studies of Drugs

Table 3 shows the data of MTT analysis of model leukemic cancer cell K562 viability after 1 and 3 days of incubation with Dox-containing formulations. Free Dox works confidently; however, its effect on cells limits the survival rate to 30%, which is insufficiently effective. At the same time, micellar non-covalent formations DoxM1-M3 demonstrate the dose-dependent nature of cytostatic activity and, at a concentration of 50 µM, reduce cell viability to 14% on the first day. It is worth considering that the drug releases from the non-covalent micelles for hours–days (Figure 5); therefore, the powerful effect of micellar cytostatics is achieved mainly on day 3: cell viability is close to 0. In the case of covalent conjugates, reduced activity (compared to free Dox) is observed on day 1 due to covalent cross-linking with the polymer and, consequently, reduced permeability to cells.

The cytostatic effect of conjugates strongly depends on the incubation time when Dox is released due to glutathione in cancer cells’ microenvironment—proof of selective action only in tumors. Covalent conjugates on day 3 revealed their potential and would demonstrate true selectivity to tumors (if the Dox was not released, there would be no cytostatic effect). The indifference of S-S cross-linked micelles was observed in relation to the model of normal HEK293T cells since pH 7.4 is maintained in the medium of normal cells, and there is no excessive amount of glutathione—there is no trigger for micelles. Indeed, the action of the covalent conjugates on normal HEK293T cells was significantly reduced compared to the free drug (Figure S3).

### 2.4. Permeability of Raji Cancer Cells to Dox-Containing Formulations

**Figure 6:**
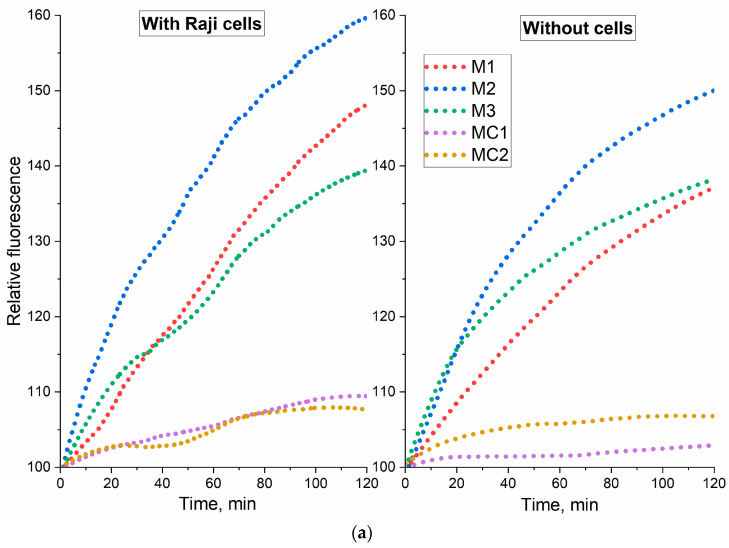

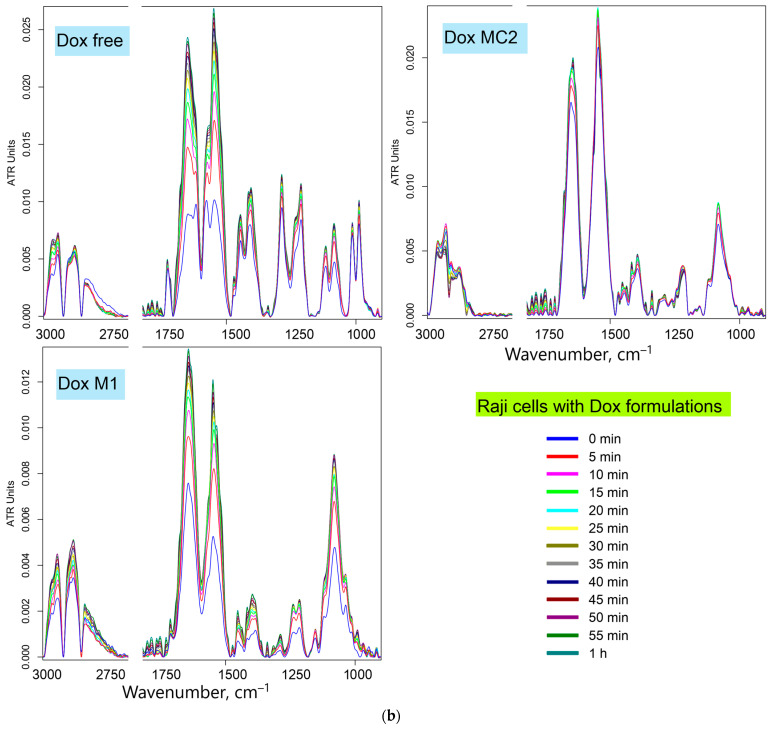
(**a**) Kinetic curves of the relative fluorescence compared to the control free Dox (sample fluorescence/free Dox fluorescence) during incubation of Dox-containing (20 µM) formulations in buffer solution and in the presence of Raji cells (10^6^ cells/mL). PBS (0.01 M, pH 7.4). T = 37 °C. λ_exci_ = 480 nm. λ_emi_ = 590 nm. (**b**) FTIR spectra of Raji cells (10^7^ cells/mL) during incubation with Dox-containing formulations. PBS (0.01 M, pH 7.4). T = 37 °C. DoxM1—Dox in Chit5-LA, DoxM2—Dox in Chit5-OA, DoxM3—Dox in Hep-OA, DoxMC1—Dox-GSSG-Chit5-OA, DoxMC2—Dox-SS-LA-Chit5.

We have shown an improved cytostatic effect of micellar formulations of Dox, as well as selectivity to tumor cells of covalent conjugates. To confirm the observed effects, we conducted a kinetic experiment (Figure 6a) to determine the permeability of Dox formulations in lymphoma cancer cells Raji. The data are given regarding the kinetics of free Dox—for convenience of interpretation: the increasing course of the curves relative to the control (free Dox) indicates the Dox release from the micelles, followed by penetration into cancer cells. Micellar formulations (DoxM1—Dox in Chit5-LA, DoxM2—Dox in Chit5-OA, DoxM3—Dox in Hep-OA) actively release Dox (in the presence of cells) and covalent conjugates (DoxMC1—Dox-GSSG-Chit5-OA, DoxMC2—Dox-SS-LA-Chit5) rather slowly, which is consistent with the data on Dox release in the presence of glutathione in the absence of the cells (Figure 5). Comparing systems with and without cells, the growth of the fluorescence signal increases to a quarter of the initial one, which indicates Dox penetration into cells and ignition of fluorescence. There are interesting observations for covalent conjugates (containing -S-S- bonds): in the absence of cells, Dox detachment does not occur (the curves slightly differ from the Dox free control), while in the presence of cancer cells, an increasing course of curves is observed, which indicates to Dox release and its gradual accumulation in Raji cells. Thus, we have shown the effectiveness of micellar conjugates against tumor cells. An interesting effect has been revealed—the ability to observe the release of drugs from micelles and penetration into cells using fluorimetry without using microscopy, while the data are well correlated. An important observation can be made by comparing heparin and chitosan micelles (green and red curves): in the presence of cells, a noticeable difference is observed for chitosan-based DoxM1-2 micelles, while in the case of heparin DoxM3 micelles, the difference is not so pronounced. This fact can be explained by the values of the zeta potentials of cells and polymers. The zeta potential (ζ-potential) of chitosan particles is positive, ranging from +7 to +10 mV for chitosan modified with oleic acid (OA) residues and from +15 to +25 mV for lipoic acid (LA)-modified chitosan. Heparin particles have a zeta potential around −20 to −30 mV. Interaction with target cells, such as cancer cells, is explained in part by electrostatic attraction. The ζ-potential of human cells at a pH of 7.5 is approximately −20 mV, making chitosan micelles more actively adsorbed onto the surface of these cells. Heparin particles can also interact with cancer cells through other mechanisms, though they interact significantly less with healthy cells. In addition, in the presence of cells, stimulus sensitivity of covalent Dox-containing conjugates is realized—significant change in the lilac curve was observed due to sensitivity to the tumor microenvironment.

### 2.5. Dox-Containing Formulation Interactions with Eukaryotic Cells: The Molecular Details

We have shown an improved cytostatic effect of micellar formulations of Dox, as well as selectivity. Regarding the cells’ permeability for the drug formulations, we have recently developed an obvious and sensitive technique based on FTIR spectroscopy [59], providing complementary information regarding the details of the drug’s interaction with individual cell components. FTIR spectroscopy is a highly informative technique that allows us to study the interaction between cells, drugs, and polymers on a molecular level. In recent years, there have been numerous studies using machine learning techniques to analyze infrared spectra, which can predict the progression of cancer or inflammation [60,61,62]. The methodology for studying cell–drug interactions and delivery systems using Fourier transform infrared spectroscopy (FTIR) was validated using control methods such as confocal microscopy, fluorescence spectroscopy, and MTT assays. Good data correlations were also revealed elsewhere [25,26,58,59]. This study demonstrates that FTIR provides valuable complementary information to fluorometry and confocal microscopy methods.

Here, to clarify the molecular details of the interactions of Dox and its micellar forms with cancer cells (Raji), we used FTIR spectroscopy (Figure 6b). Characteristic peaks corresponding to oscillations in the bonds of the components of the lipid bilayer (3000–2850 cm^−1^), proteins (amide I 1700–1600 cm^−1^, amide II 1600–1500 cm^−1^), carbohydrate fragments (1100–1000 cm^−1^) appear in the FTIR spectra of cells. The penetration of Dox into cells is accompanied by an increase in peak intensity: amide I is analytically significant. In the case of free Dox, there is a 2.5-fold increase in the intensity of amide I per hour. In the case of micellar non-covalent Dox, there is a 2-fold increase and small 15–20% change for the covalent conjugate, which is consistent with the data in Figure 6a. The interaction of micelles with the cell membrane is accompanied by an increase in peak intensity at 1100–1000 cm^−1^. The greatest change is observed in the case of the non-covalent micellar (only in this combination) formation of DoxM1 since the structure of non-cross-linked micelles is looser than that of covalent links. However, Dox in chitosan micelles intercalates more effectively into DNA (band 1300–1200 cm^−1^) than in the case of a simple Dox. At the same time, if the drug weakly penetrates and interacts (as in the case of covalent micelles DoxMC1-2), then small changes are observed—control of the validity of the method. Thus, using FTIR spectroscopy, we have shown that non-covalent micellar Dox penetrates cancer cells efficiently, and the cleavage of Dox from the covalent conjugate is slowed, but it is necessary for prolongation and selectivity.

To prove the selectivity of the action of micelles on eukaryotic cells, the authors used normal HEK293T cells as a control (Figure S3). At 37 °C, Dox effectively penetrates cells (we observe an almost 3-fold increase in the intensity of amide I), while micellar DoxM1 penetrates much weaker (less than 10% of the change in spectra). Thus, micellar formations show selectivity to cancer cells.

### 2.6. Confocal Visualization of Micellar Formulations Based on Doxorubicin Action on Cancer Cells

**Figure 7:**
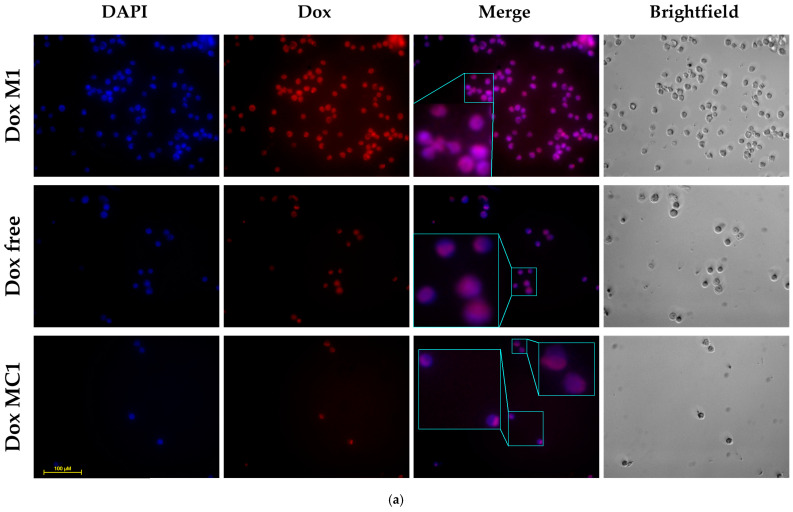

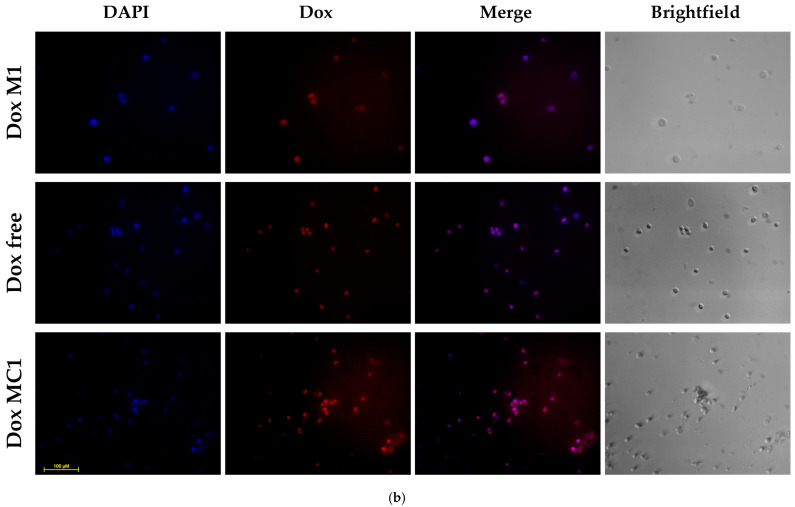
Fluorescence images of (**a**) A875 cells and (**b**) K562 cells after 2 h incubation with Dox-containing formulations. C_Dox_ = 2 μM. The nuclei are stained with DAPI (1 μg/mL). DAPI channel: λ_exci_ = 310–380 nm, λ_emi_ = 420–500 nm. Dox channel: λ_exci_ = 500–560 nm, λ_exci_ = 590–700 nm. The scale segment is 100 µm. DoxM1—Dox in Chit5-LA, DoxM2—Dox in Chit5-OA, DoxM3—Dox in Hep-OA, DoxMC1—Dox-GSSG-Chit5-OA, DoxMC2—Dox-SS-LA-Chit5.

One of the highly informative methods for visualizing the effect of cytostatics on cells is confocal laser scanning microscopy (CLSM). Figure 7a,b shows fluorescent images of cells A875 and K562 incubated with Dox formulations: micellar non-covalent Dox and micellar covalent Dox in comparison with free Dox. In the case of A875 cells, the best permeability is achieved for DoxM1, while covalent doxorubicin penetrates relatively weakly (during the incubation time of 2 h). On the contrary, in the case of the leukemia cell K562, we observed high efficiency of both micellar formulations and the covalent conjugate of Dox, which demonstrated the highest efficiency. The accumulation of Dox in the nucleus of cancer cells is a characteristic feature of this cytostatics, and this is observed in the case of free Dox and covalent conjugate DoxMC1 (purple and blue colors in the Merge channel). On the contrary, non-covalent micellar DoxM1, due to its high adsorption on the cell surface, causes a predominantly purple color (wider area coverage) in the Merge channel (zoom in Figure 7a).

**Figure 8:**
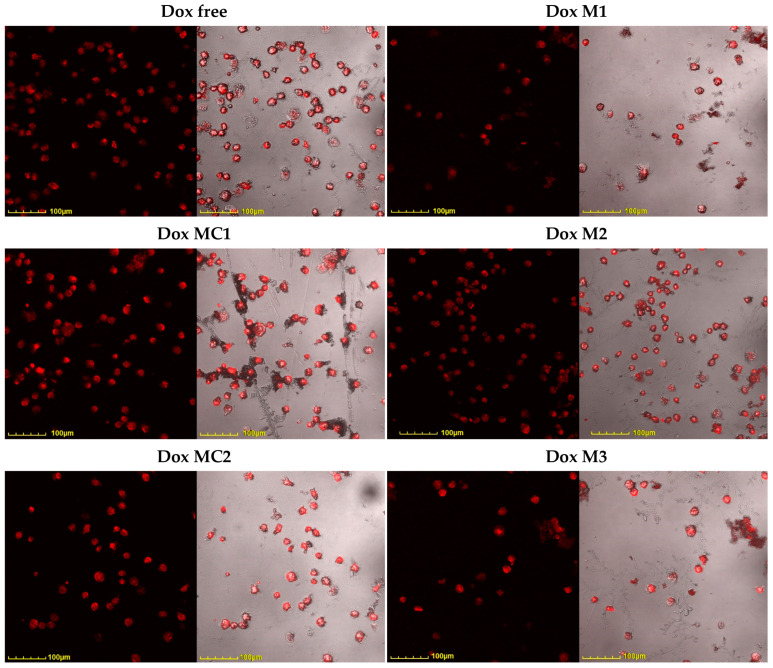
Confocal laser scanning fluorescence images of Raji cells after 2 h incubation with Dox-containing formulations. C_Dox_ = 10 μg/mL. λ_exci_ = 488 nm, λ_emi_ = 570–730 nm. The scale segment is 100 µm. DoxM1—Dox in Chit5-LA, DoxM2—Dox in Chit5-OA, DoxM3—Dox in Hep-OA, DoxMC1—Dox-GSSG-Chit5-OA, DoxMC2—Dox-SS-LA-Chit5.

Figure 8 shows confocal images of Raji cells pre-incubated with all the studied formulations. The greatest efficiency of Dox penetration into cancer cells is achieved in the case of micellar formulations: covalent (DoxMC1-2) or heparin-based (DoxM3). Taking into account the prolonged release of Dox, this is a significant result. That is, in the case of micellar cytostatics, the bioavailability of the drug is significantly higher, while penetration into healthy cells is reduced, as we have shown earlier [26].

**Figure 9:**
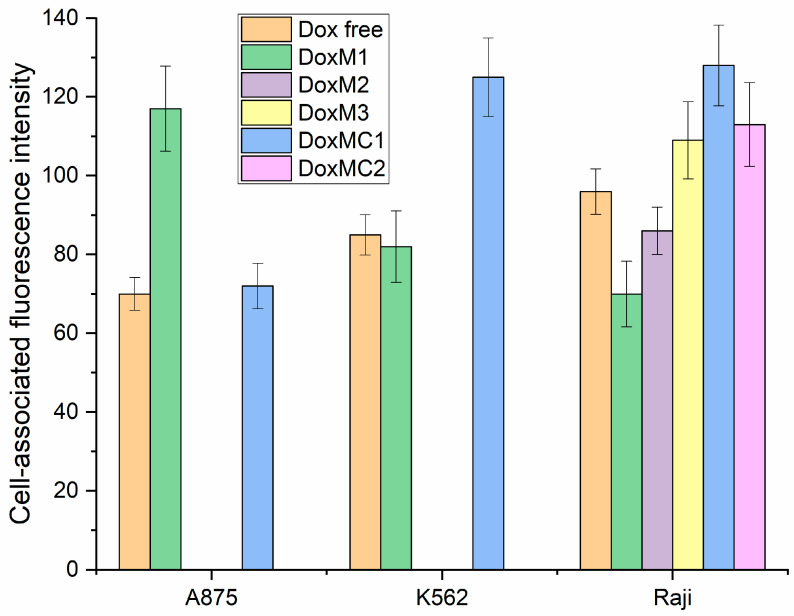
Cell-associated Dox fluorescence values depending on the composition of the Dox-containing formulation (10 μg/mL). Determined by fluorescent image analysis and fluorescence quantification of Dox uptake. PBS (0.01 M, pH 7.4). T = 37 °C. DoxM1—Dox in Chit5-LA, DoxM2—Dox in Chit5-OA, DoxM3—Dox in Hep-OA, DoxMC1—Dox-GSSG-Chit5-OA, DoxMC2—Dox-SS-LA-Chit5.

Quantitative data on the permeability of Dox-containing formulations are presented in Figure 9. Heparin micelles with Dox (DoxM3, which also showed better activity according to the MTT test), as well as covalent conjugates (DoxMC1-2), penetrate most actively. At the same time, it is worth noting that incubation was carried out for 2 h (conditions to identify differences between the formulations); therefore, covalent DoxMC1-2 has not yet revealed its potential, which could be realized after 48–72 h. Micrographs of Raji and K562 cells show that almost all of the Dox-containing samples were completely absorbed. Absorption occurs partially with polymeric particles since their size is 200–250 nm—internalization into phagocytic Raji and K562 cells. This means that the effectiveness of cytostatic accumulation in the tumor is retained, and selectivity will be ensured compared to healthy cells.

At the same time, in the example of epithelial A875 cells, the penetration of polymeric particles is noticeably much less since they are surface-attached and non-phagocytic.

So, the methodology for studying cell–drug interactions and delivery systems using Fourier transform infrared spectroscopy (FTIR) was validated using control methods such as confocal microscopy, fluorescence spectroscopy, and MTT assays. The resulting efficiency for the studied samples is shown in Table 4. Data on the effectiveness of Dox penetration into cancer cells in the short term, obtained by FTIR spectroscopy, fluorometry, and MMT test, makes it possible to arrange these formations in a sequence in descending order of permeability: DoxM2 ≈ DoxM3 ≥ Dox ≥ DoxM1 >> DoxMC1, DoxMC2. While FTIR spectroscopy is a valuable tool for studying the precise interactions between cells, drugs, and polymers, confocal microscopy shows a similar range of effectiveness visually. Good data correlations between the results obtained by the different methods were revealed.

## 3. Conclusions

The paper considers an approach to the creation of the cytostatic drug carrier (Dox), which is stable in blood circulation and disintegrates after intracellular uptake in leukemic cells. Polymeric micelles based on chitosan, heparin, and fatty acid residues (lipoic or oleic) are characterized by pH-, thermo-, and glutathione sensitivity to the tumor microenvironment. In a weakly acidic environment, protonation of chitosan amino groups occurs, resulting in the loosening of the micelle structure and release of the drug into cancer cells. We have obtained both non-covalent micellar formulations with doxorubicin (Dox) and covalent Dox-SS-polymer compounds in which the Redox-sensitive disulfide bond is a trigger to the tumor environment. Using fluorescence and FTIR spectroscopy, we have shown the prolonged nature of the release of Dox from the nanoparticles. At the same time, it was found that the release rate increases up to 6 times in the presence of glutathione as a model substance in the tumor environment. For Dox-SS-polymer conjugates in the absence of cells, Dox detachment does not occur, while in the presence of cancer cells, Dox release and its gradual accumulation in Raji cells were observed—a direct indication of the stimuli-sensitivity of micelles. Using FTIR spectroscopy, the molecular details of the interaction of Dox-containing formulations with eukaryotic cells were determined, and the selectivity of the action of micellar Dox against cancer cells Raji vs. normal cells HEK293T was shown. Using confocal microscopy, the penetration of Dox-containing formulations into cancer cells of two types was visualized: phagocytic cells, capable of absorbing large particles such as micelles—Raji and K562; and skin cancer cells A875 (epithelial, non-phagocytic cells), weakly absorbing large polymer particles. We regard the most optimal formulations for the treatment of leukemia as Dox in Hep-OA and Dox-GSSG-Chit5-OA. Chitosan micelles, both covalent and non-covalent, can be used to treat skin cancer. With enhanced metabolism of cancer cells and the tendency to absorb the large particles, tumors maintain high levels of glutathione and a slightly acidic environment, but at the same time, this is the weak point. This was taken advantage of when using “smart” stimuli-sensitive micelles—by implementing the “Aikido principle”.

## 4. Materials and Methods

### 4.1. Reagents

In this work, the following chemicals were used: chitosan 5 kDa (Chit5), heparin 20–30 kDa (Hep), oleic acid (OA), lipoic acid (LA), 1-Ethyl-3-(3-dimethylaminopropyl) carbodiimide (EDC), N-hydroxysuccinimide (NHS), doxorubicin (Dox) hydrochloride from Sigma Aldrich (St. Louis, MO, USA). Dithiothreitol, acid, solvents, salts, and others were Reachim production (Moscow, Russia).

### 4.2. The Synthesis and Characterization of Amphiphilic Polymers and Dox-Containing Micelles

#### 4.2.1. Heparin and Chitosan Grafted Conjugates Synthesis

*Chit5-OA* and *Chit5-LA*. The oleic acid (OA) and lipoic acid (LA) 30 mg were dissolved in 5 mL CH_3_CN/PBS (4:1 *v*/*v*, pH 7.4). A 2.5-fold molar excess of EDC and a 1.3-fold molar excess of NHS were added to DMF. Acid activation was performed for 20 min at 50 °C. Then, pre-dissolved Chit5 (90 mg in 10 mL 1 mM HCl, followed by pH adjustment to 7) was added to the reaction mixtures. Then, the mixtures were incubated for 6 h at 50 °C. The reaction mixtures were purified using centrifuge filters (3 kDa, 10,000× *g*, 3 × 10 min), then dialyzed against water for 12 h (cut-off 6–8 kDa). The Chit5 modification degree was estimated from a spectrophotometric titration technique using 2,4,6-trinitrobenzenesulfonic acid in sodium-borate buffer (pH 9.2).

*Hep-OA*. 125 mg of heparin (Hep) was dissolved in 10 mL of PBS. A 2.5-fold molar excess (in relation to the amount of oleylamine) of EDC and a 1.3-fold molar excess (in relation to the amount of oleylamine) of NHS was added to DMF. Hep activation was performed for 20 min at 50 °C. Then, pre-dissolved oleylamine (40 mg in 5 mL CH_3_CN/PBS (4:1 *v*/*v*, pH 7.4)) was added to the reaction mixture followed by incubation for 6 h at 50 °C. The reaction mixtures were purified using centrifuge filters (10 kDa, 10,000× *g*, 3 × 10 min), then dialyzed against water for 12 h (cut-off 12–14 kDa).

All samples were freeze-dried at −60 °C (Edwards 5, BOC Edwards, Crawley, UK).

#### 4.2.2. Non-Covalent Dox Micellar Formulation Synthesis

*DoxM1-M3*. Chit5-LA, Chit5-OA, and Hep-OA were dissolved in PBS (0.01 M, pH 7.4) at a concentration of 10 mg/mL. Dox solution (2 mg/mL) was added to these solutions until the loading degrees indicated in Table 1 were reached ±1%. Micelle samples were prepared by probe-type ultra-sonic treatment (snow, 10 min) followed by extrusion through a 200 nm membrane.

#### 4.2.3. Covalent Dox Micellar Formulation Synthesis

*Dox-GSSG*. Oxidized glutathione was incubated with 3-mol equivalents of 4-hydroxybenzaldehyde in the presence of 5 eq. NaBH_3_CN at pH 5 (sodium acetate buffer) for 2 h at 60 °C. Then, 1 mL of 3% H_2_O_2_ was added to the mixture. The mixture was purified using centrifuge filters (3 kDa, 10,000× *g*, 5 min). Dox was incubated for 3 h at 40 °C in PBS with protected GSSG (1:1.2 mol/mol) in the presence of 2.5-fold molar excess of EDC and 1.3-fold molar excess of NHS. Dox-GSSG was purified using dialysis against water for 12 h (cut-off 1 kDa).

*DoxMC1* (Dox-GSSG-Chit5-OA). Chit5-OA was incubated for 4 h at 40 °C in PBS with Dox-GSSG (1:2 mol/mol) in the presence of 2.5-fold (on Dox) molar excess of EDC and 1.3-fold molar excess of NHS. Dox-GSSG-Chit5-OA was purified using dialysis against water for 12 h (cut-off 6–8 kDa).

*DoxMC2* (Dox-SS-LA-Chit5). Chit5-LA was incubated for 2 × 30 min at 60 °C in PBS with Dox-GSSG (1:2 mol/mol) in the presence of (1) 3-fold molar excess of dithiothreitol, (2) 10-fold molar excess of H_2_O_2._ Dox-SS-LA-Chit5 was purified using dialysis against water for 12 h (cut-off 6–8 kDa).

All samples were freeze-dried at −60 °C (Edwards 5, BOC Edwards, Crawley, UK). Micelle samples were prepared by probe-type ultra-sonic treatment (snow, 10 min) followed by extrusion through a 200 nm membrane.

### 4.3. Characterization of Polymers and Micelles

FTIR spectra of samples were registered using an FTIR microscope MICRAN-3 and Bruker Tensor 27 spectrometer equipped with a liquid-nitrogen-cooled MCT (mercury cadmium telluride) detector, as described earlier [26,63].

^1^H spectra of samples were registered using a Bruker Avance 400 spectrometer (Bruker, Ettlingen, Germany) with an operating frequency of 400 MHz: Figure S5.

Circular dichroism spectra were recorded on Jasco J-815 CD Spectrometer (JASCO, Tokyo, Japan) and were used to estimate the deacetylation degree in Chit5, which amounted to (92 ± 3)%.

Atomic force microscopy (AFM microscope NTEGRA II, NT-MDT Spectrum Instruments, Moscow, Russia) was used to visualize polymeric micelles based on grafted chitosan and compare it in terms of shape and size with non-modified chitosan.

### 4.4. Determination of Dox Loading Degree into Micelles and Release Kinetics

The amount of Dox loaded in micellar formulations was determined by absorption at 488 nm and fluorescence intensity at 590 nm.

The release experiment was structured as follows: 1 mL of the Dox-containing solution (1 mg/mL) was placed inside a dialysis bag with a cut-off weight of 7 kDa, and then the bag was placed in an external solution PBS (10 mL, 0.01 M, pH 7.4). The system was incubated at 37 °C, and samples were taken in which Dox was determined fluorimetrically.

UV–vis spectra of solutions were recorded on the AmerSham Biosciences UltraSpec 2100 pro device (Cambridge, UK). Fluorescence of Dox was measured using a Varian Cary Eclipse spectrofluorometer (Agilent Technologies, Santa Clara, CA, USA) at 22 °C: λ_exci_ = 490 nm, λ_emi_ = 560 nm.

### 4.5. Cell Cultivation and Toxicity Assay

K562 leukemia cells, A875 melanoma cells, Raji lymphoblast-like cells, and linear cells of the embryonic kidney human epithelium HEK293T were obtained from the Lomonosov Moscow State University Depository of Live Systems Collection (Moscow, Russia). Cells were grown in RPMI-1640 medium (Gibco, Thermo Fisher Scientific Inc., Waltham, MA, USA) supplemented with 5% fetal bovine serum (Capricorn Scientific, Ebsdorfergrund, Germany) and 1% Na-pyruvate (Paneco, Moscow, Russia) at 5% CO_2_/95% air in a humidified atmosphere at 37 °C.

### 4.6. FTIR Spectroscopy as a Tool for Studying Dox Interaction with Cells

Cell suspensions (3–5 × 10^6^ cells/mL) were washed twice with sterile PBS (pH = 7.4) from the culture medium by centrifuging (Eppendorf centrifuge 5415C, 2 × 5 min, 4000× *g*).

Cells were precipitated, followed by resuspension in PBS to a concentration 1 × 10^7^ cells/mL. 20 µL of cell suspension was placed on a spectrometer chamber, 10 µL of Dox-containing preparation was added (1 mg/mL according to Dox), the samples were incubated at 37 °C, and spectra were recorded in increments of 5–10 min. Absorbed by cells and free Dox were quantified using fluorescence spectroscopy.

### 4.7. Confocal Laser Scanning Microscopy for Visualization of Dox Interaction with Cells

Cells were precipitated as described above, followed by 2 h incubation with Dox-containing formulations (10 μg/mL on Dox). The cells were washed twice with PBS (5 min, 4000× *g*), followed by placing them in 96-well tablet cells and treating them with formaldehyde. Confocal images were recorded on the confocal laser scanning microscope (CLSM) Olympus FluoView FV1000, which was equipped with both a spectral version scan unit with emission detectors and a transmitted light detector. The scan area was 80 × 80 µm^2^. Olympus FV10 ASW 1.7 software was used for the acquisition of the images.

### 4.8. Statistical Analysis

Statistical analysis of cytotoxicity and spectral data was performed using the Student’s *t*-test Origin 2022 software (OriginLab Corporation, Northampton, MA, USA). Values are given as the mean ± SD of three or five experiments.

## Figures and Tables

**Table 1:** Designations and characteristics of polymers and Dox-containing formulations based on polymeric micelles.

Dox Containing Micellar Formulation		Dox Mass Percentage, %	Average Mw of One Polymeric Structure Unit, kDa	Critical Micelle Concentration ***, nM	Ref.	
Brief Designation *	Chemical Composition **				Synthesis	FTIR Spectra
*DoxM1*	Dox in Chit5-LA	13 ± 1	6.2 ± 0.4	7 ± 1 (without Dox, 6 ± 1)	Figure 1a	Figure S1 and Figure 3a
*DoxM2*	Dox in Chit5-OA	10 ± 1	6.6 ± 0.5	8 ± 2 (without Dox, 4 ± 1)		Figures 2a and 3a
*DoxM3*	Dox in Hep-OA	5.8 ± 0.3	23 ± 5	15 ± 2 (without Dox, 12 ± 3)		Figures 2b and 3a
*DoxMC1*	Dox-GSSG-Chit5-OA	13 ± 1	8.3 ± 0.7	14 ± 3	Figure 1b,c	Figure 3b,c
*DoxMC2*	Dox-SS-LA-Chit5	14 ± 1	7.5 ± 0.6	9 ± 1	Figure 1b,d	Figure 3b,d

* M means micellar, MC means micellar covalent. ** Chit5—chitosan 5 kDa, Hep—heparin 20 kDa, LA—lipoic acid residue, OA—oleic acid residue, GSSG—reduced glutathione residue. *** Critical micelle concentrations were determined using the pyrene-probe technique.

**Table 2:** Kinetic parameters of Dox release: initial rates, accumulated release concentrations after 7 h. Dialysis method in an external solution (1 to 10 by volume, membrane with a cut-off mass of 7 kDa). C_Dox_ = 1 mg/mL. PBS (0.01 M, pH 7.4). T = 37 °C. For Dox-free and Dox M1-M3, the release rate was practically independent of the GSH concentration (no more than 10%).

Dox Formulation	Initial Rate, %/h	Accumulated Concentration after 7 h, %
Dox-free	88 ± 5	100
DoxM1	5 ± 1	19 ± 2
DoxM2	11 ± 2	22 ± 3
DoxM3	29 ± 4	64 ± 5
DoxMC1	8 ± 1 (0 mM GSH) 10 ± 1 (1 mM GSH) 37 ± 4 (10 mM GSH)	16 ± 3 (0 mM GSH) 25 ± 4 (1 mM GSH) 64 ± 7 (10 mM GSH)
DoxMC2	8 ± 1 (0 mM GSH) 16 ± 2 (1 mM GSH) 25 ± 2 (10 mM GSH)	13 ± 2 (0 mM GSH) 28 ± 3 (1 mM GSH) 54 ± 5 (10 mM GSH)

**Table 3:** K562 cells viability MTT assay. Cells were treated with Dox-containing formulation: 5 and 50 µM. RPMI-1640 medium supplemented with 5% fetal bovine serum and 1% sodium pyruvate at 5% CO_2_/95% air in a humidified atmosphere at 37 °C. DoxM1—Dox in Chit5-LA, DoxM2—Dox in Chit5-OA, DoxM3—Dox in Hep-OA, DoxMC1—Dox-GSSG-Chit5-OA, DoxMC2—Dox-SS-LA-Chit5.

Dox Formulation	C_Dox_ = 50 µM		C_Dox_ = 5 µM	
	1 Day	3 Day	1 Day	3 Day
*Dox-free*	30 ± 3	4.3 ± 0.5	33 ± 2	5 ± 1
*Dox M1*	31 ± 2	<1	43 ± 4	3.3 ± 0.3
*Dox M2*	26 ± 3		40 ± 5	4.1 ± 0.7
*Dox M3*	14 ± 1		23 ± 1	2.9 ± 0.2
*Dox MC1*	54 ± 5	6.4 ± 1.2	69 ± 8	29 ± 5
*Dox MC2*	57 ± 8	5.3 ± 1.1	81 ± 6	21 ± 3

**Table 4:** The resulting schematic characteristics of Dox-containing formulations based on polymeric micelles in terms of tumor targeting. “++” means a bright effect, “+” means a good effect, “ ± ” means a weak effect, “–+” means a very weak effect, “–” there is no effect.

Dox Containing Micellar Formulation		Permeability to Eukaryotic Cells				Toxicity to Eukaryotic Cells		Tumor-Sensitivity	
Brief Designation	Chemical Composition	Cancer K562	Cancer Raji	Cancer A875	Normal HEK293T	Cancer K562	Normal HEK293T	pH 5.5–6.5	Glutathione
*Dox*	Dox free	+	+	±	+	+	+	–	–
*DoxM1*	Dox in Chit5-LA	++	±	+	±	++	±	+	+
*DoxM2*	Dox in Chit5-OA	+	+	+	±	++	±	+	–
*DoxM3*	Dox in Hep-OA	++	++	+	±	++	±	±	–
*DoxMC1*	Dox-GSSG-Chit5-OA	+	++	++	–	+	–+	+	++
*DoxMC2*	Dox-SS-LA-Chit5	+	++	+	–	+	–+	+	++

## Data Availability

The data presented in this study are available in the main text and Supplementary Materials.

## Supplementary Materials

The following supporting information can be downloaded at https://www.mdpi.com/article/10.3390/gels10030157/s1, Figure S1: FTIR spectra of Chit5, LA, its conjugate Chit5-LA. PBS (0.01 M, pH 7.4). T = 22 °C. Figure S2: Zoomed FTIR spectra of Chit5-LA and DoxMC2 (Dox-SS-LA-Chit5) in the wavenumber region 2650–2450 cm^−1^. PBS (0.01 M, pH 7.4). T = 22 °C. Figure S3: FTIR spectra of normal HEK293T cells during incubation for 30 min with free Dox (0.1 mg/mL) and micellar Dox formulation (DoxM1). PBS (0.01 M, pH = 7.4). T = 22 or 37 °C. Figure S4: Normalized fluorescence emission spectra of pyrene probe for determination of the critical micelle concentration (CMC) of Chit5-OA and calculation of hydrophobic–hydrophilic balance in polymeric micelles. PBS (0.01 M, pH 7.4). λ_exci_ = 340 nm. T = 22 °C. Figure S5: ^1^H NMR of Chit5 grafted with (a) lipoic acid and (b) oleic acid. PBS (0.01 M, pH = 7.4). T = 22 °C.

## Abbreviations

Chit	Chitosan
CLSM	confocal laser scanning microscopy
Dox	Doxorubicin
EDC	1-Ethyl-3-(3-dimethylaminopropyl) carbodiimide
FTIR	Fourier transformed infrared
GSH	reduced glutathione
GSSG	oxidized glutathione
Hep	heparin
LA	lipoic acid
NHS	N-hydroxysuccinimide
OA	oleic acid
