# Supplementary material for: Disulfide Cross-Linked Polymeric Redox-Responsive Nanocarrier Based on Heparin, Chitosan and Lipoic Acid Improved Drug Accumulation, Increased Cytotoxicity and Selectivity to Leukemia Cells by Tumor Targeting via “Aikido” Principle
Source: Gels. 2024 Feb 20;10(3):157. doi: 10.3390/gels10030157 (PMC10970284; doi:10.3390/gels10030157)
Supplement: Supplementary file 1 [file gels-10-00157-s001.zip › gels-2873966-supplementary.pdf]

Disulfide cross-linked polymeric redox-responsive nanocarrier based on heparin, chitosan and lipoic acid improved drug accumulation, increased cytotoxicity and selectivity against leukemia cells by tumor targeting via “Aikido” principle

Igor D. Zlotnikov [1], Alexander A. Ezhov [2], Natalia V. Dobryakova [1] and Elena V. Kudryashova [1,*]

[1] Faculty of Chemistry, Lomonosov Moscow State University, Leninskie Gory, 1/3, 119991 Moscow, Russia; zlotnikovid@my.msu.ru (I.D.Z.);
[2] Faculty of Physics, Lomonosov Moscow State University, Leninskie Gory, 1/2, 119991 Moscow, Russia; alexander-ezhov@yandex.ru
* Correspondence: helenakoudriachova@yandex.ru (E.V.K.)

Figure S1. FTIR spectra of Chit5, LA, its conjugate Chit5-LA. PBS (0.01 M, pH 7.4). T = 22 °C.

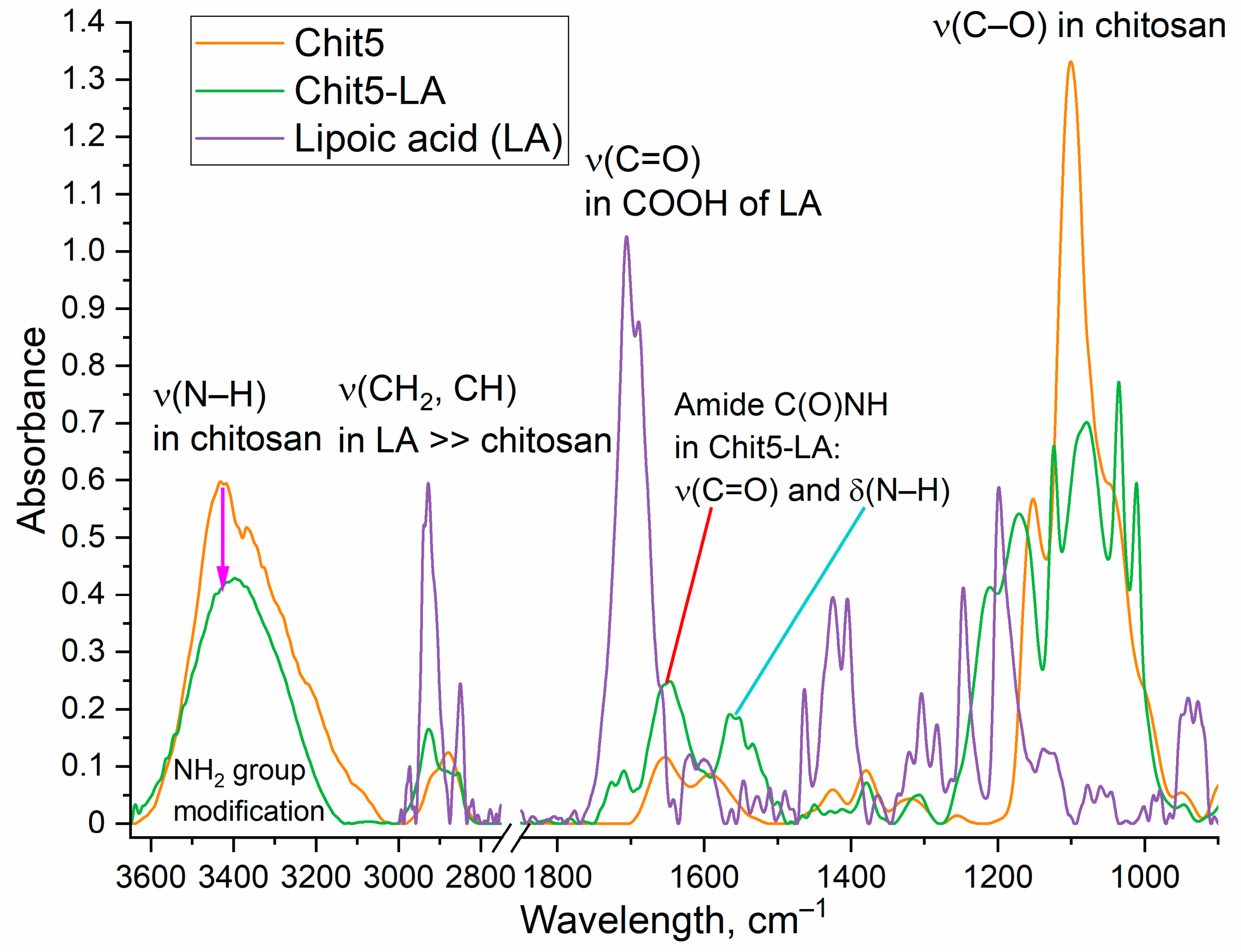

Figure S2. Zoomed FTIR spectra of Chit5-LA and DoxMC2 (Dox-SS-LA-Chit5) in the wavenumber region 2650-2450 cm^{-1}. PBS (0.01 M, pH 7.4). T = 22 °C.

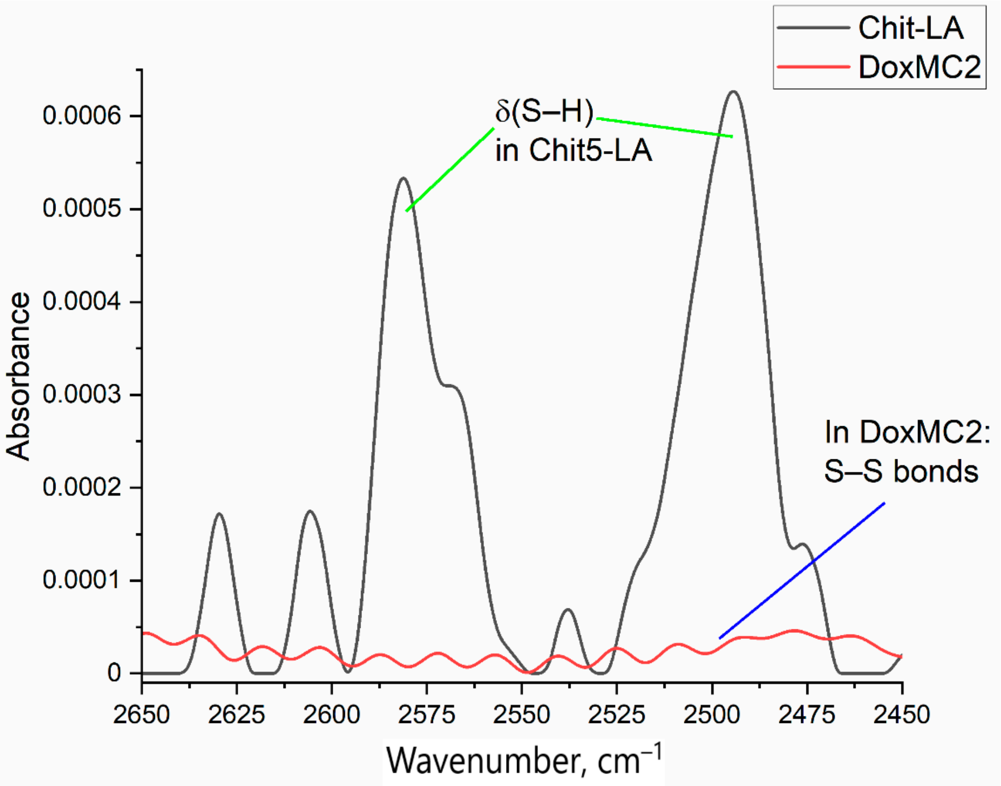

Figure S3. FTIR spectra of normal HEK293T cells during incubation for 30 min with free Dox (0.1 mg/mL) and micellar Dox formulation (DoxM1). PBS (0.01 M, pH = 7.4). T = 22 or 37 °C.

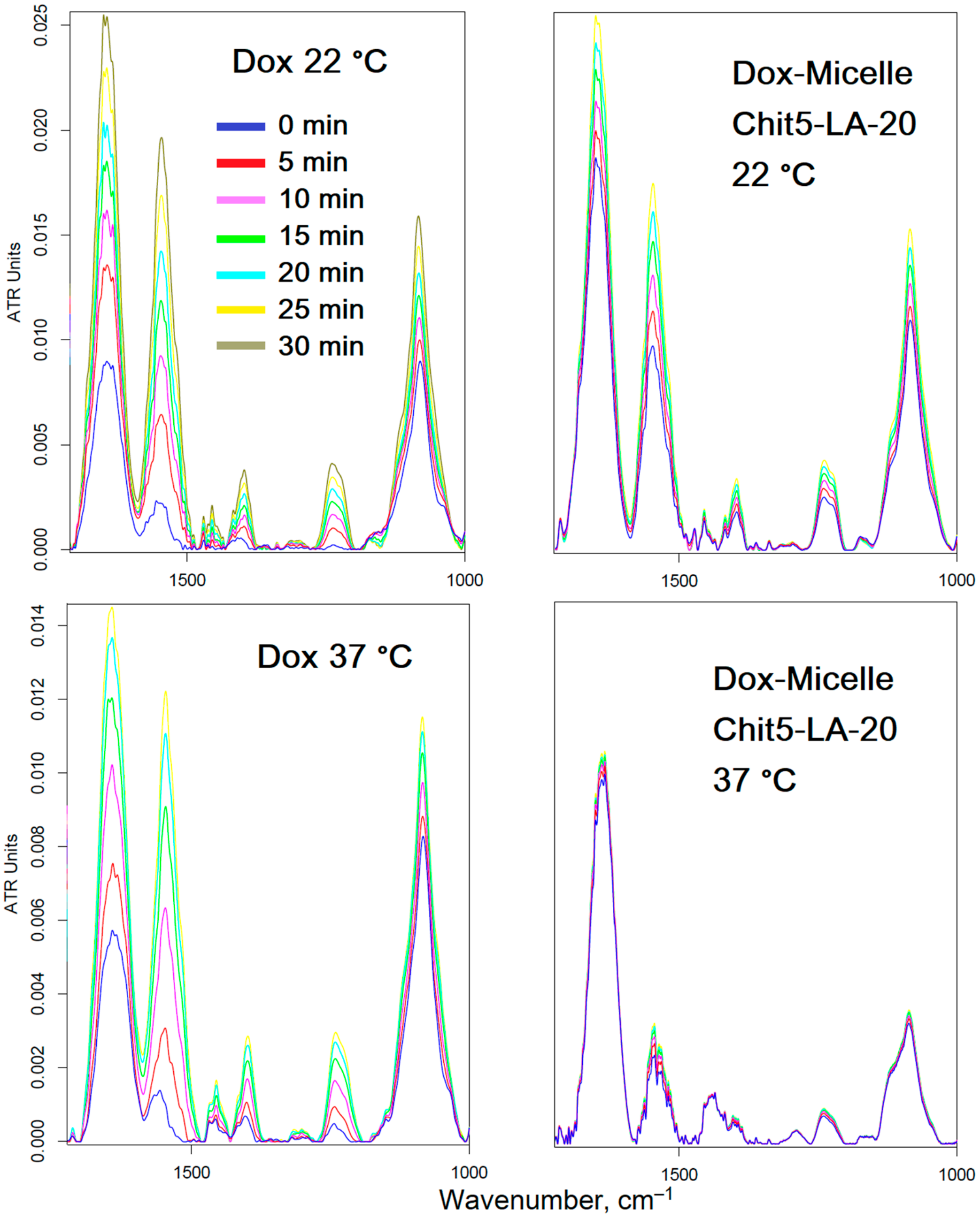

Figure S4. Normalized fluorescence emission spectra of pyrene probe for determination of the critical micelle concentration (CMC) of Chit5-OA and ccalculation of hydrophobic-hydrophilic balance in polymeric micelles. PBS (0.01 M, pH 7.4). λ_{exci} = 340 nm. T = 22 °C.

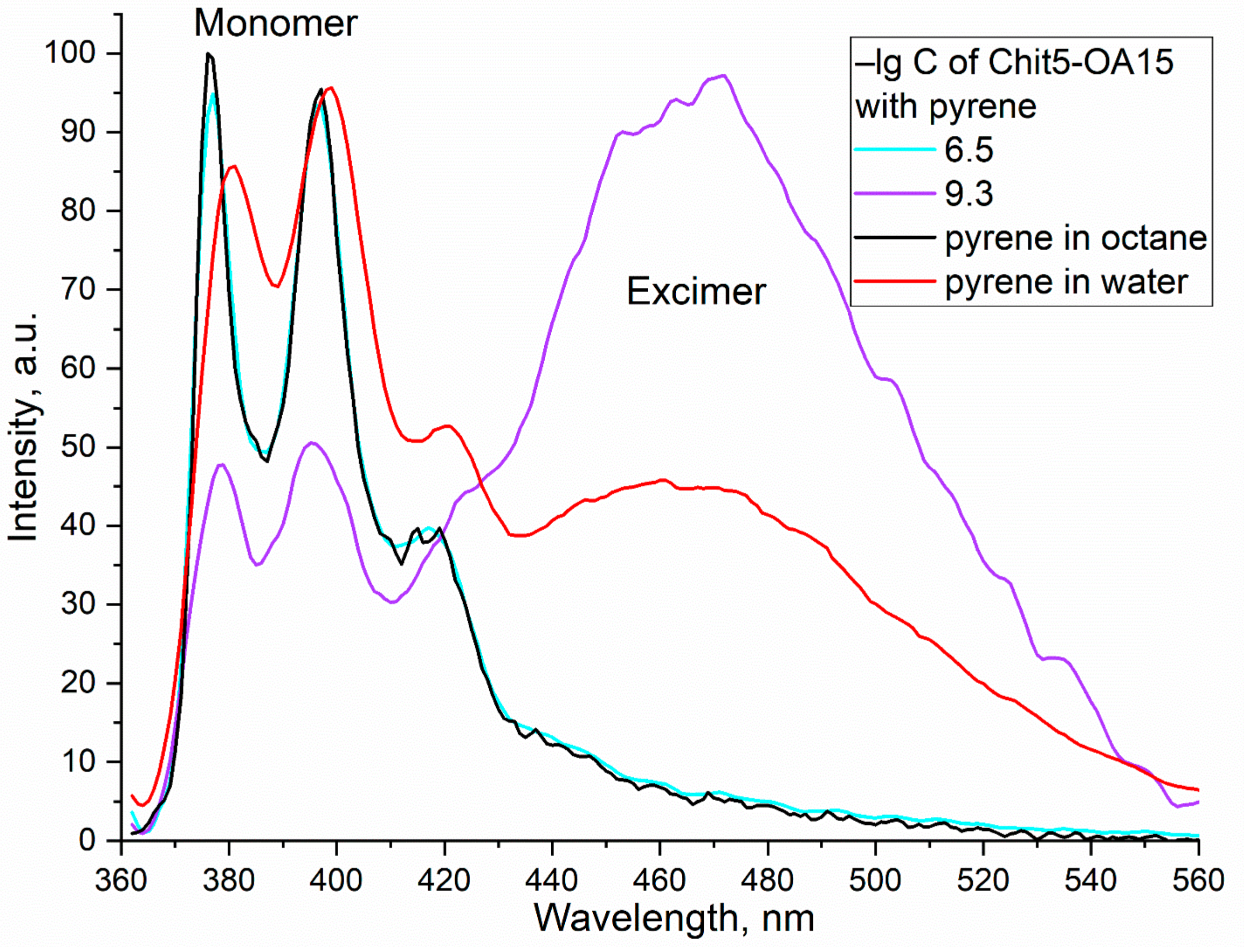

Figure S5. ^{1}H NMR of Chit5 grafted with (a) lipoic acid and (b) oleic acid. PBS (0.01M, pH = 7.4). T = 22 °C.

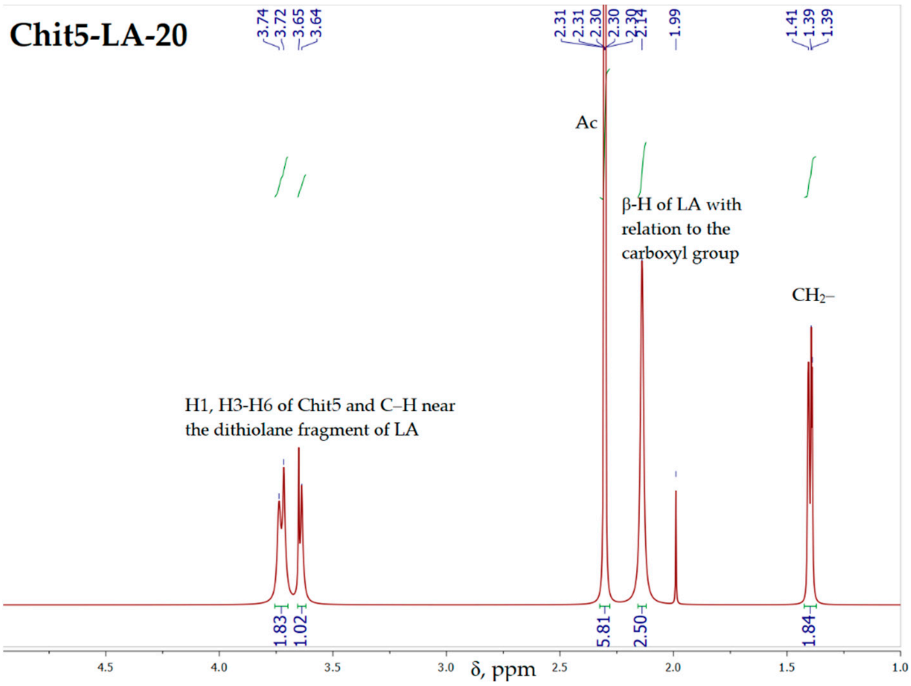

(a)

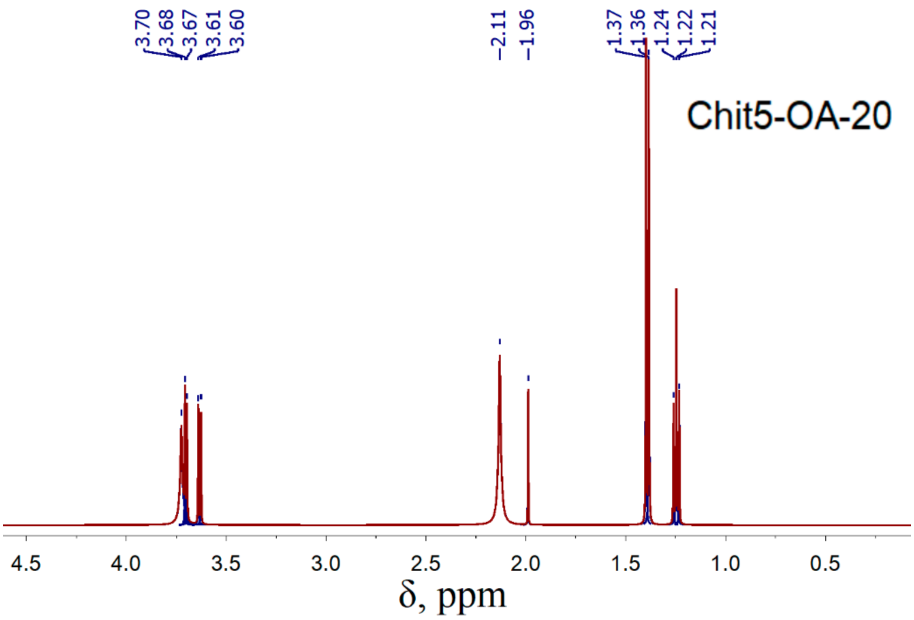

(b)
